# Effect of Calcination Temperature on Structural, Morphological and Optical Properties of Copper Oxide Nanostructures Derived from *Garcinia mangostana* L. Leaf Extract

**DOI:** 10.3390/nano12203589

**Published:** 2022-10-13

**Authors:** Yu Bin Chan, Vidhya Selvanathan, Lai-Hock Tey, Md. Akhtaruzzaman, Farah Hannan Anur, Sinouvassane Djearamane, Akira Watanabe, Mohammod Aminuzzaman

**Affiliations:** 1 Department of Chemical Science, Faculty of Science, Universiti Tunku Abdul Rahman (UTAR), Kampar Campus, Jalan Universiti, Bandar Barat, Kampar 31900, Perak, Malaysia; 2 Solar Energy Research Institute (SERI), Universiti Kebangsaan Malaysia (UKM), Bangi 43600, Selangor, Malaysia; 3 Department of Chemical Sciences, Faculty of Science and Technology, Universiti Kebangsaan Malaysia (UKM), Bangi 43600, Selangor, Malaysia; 4 Department of Biomedical Science, Faculty of Science, Universiti Tunku Abdul Rahman (UTAR), Kampar Campus, Jalan Universiti, Bandar Barat, Kampar 31900, Perak, Malaysia; 5 Institute of Multidisciplinary Research for Advanced Materials (IMRAM), Tohoku University, Sendai 980-8577, Japan; 6 Centre for Photonics and Advanced Materials Research (CPAMR), Universiti Tunku Abdul Rahman (UTAR), Sungai Long Campus, Jalan Sungai Long, Bandar Sungai Long, Kajang 43000, Selangor, Malaysia

**Keywords:** calcination, copper oxide, *Garcinia mangostana* L., green synthesis, nanoparticles

## Abstract

Synthesis of copper oxide (CuO) nanostructures via biological approach has gained attention to reduce the harmful effects of chemical synthesis. The CuO nanostructures were synthesized through a green approach using the *Garcinia mangostana* L. leaf extract and copper (II) nitrate trihydrate as a precursor at varying calcination temperatures (200–600 °C). The effect of calcination temperatures on the structural, morphological and optical properties of CuO nanostructures was studied. The red shifting of the green-synthesized CuO nanoparticles’ absorption peak was observed in UV-visible spectrum, and the optical energy bandgap was found to decrease from 3.41 eV to 3.19 eV as the calcination temperatures increased. The PL analysis shown that synthesized CuO NPs calcinated at 500 °C has the maximum charge carriers separation. A peak located at 504–536 cm^−1^ was shown in FTIR spectrum that indicated the presence of a copper-oxygen vibration band and become sharper and more intense when increasing the calcination temperature. The XRD studies revealed that the CuO nanoparticles’ crystalline size was found to increase from 12.78 nm to 28.17 nm, and dislocation density decreased from 61.26 × 10^14^ cm^−1^ to 12.60 × 10^14^ cm^−1^, while micro strain decreased from 3.40 × 10^−4^ to 1.26 × 10^–4^. From the XPS measurement, only CuO single phase without impurities was detected for the green-mediated NPs calcinated at 500 °C. The morphologies of CuO nanostructures were examined using FESEM and became more spherical in shape at elevated calcination temperature. More or less spherical nanostructure of green-mediated CuO calcinated at 500 °C were also observed using TEM. The purity of the green-synthesized CuO nanoparticles was evaluated by EDX analysis, and results showed that increasing calcination temperature increases the purity of CuO nanoparticles.

## 1. Introduction

Semiconductor metal oxide nanostructures, such as zinc oxide, titanium dioxide, nickel oxide, copper oxide (CuO) etc. play a vital role in various areas [1,2,3]. They are the building blocks of next generation technology with many industrial sectors as they have wide range of morphology, size and structures with unique physicochemical and biological properties [4,5]. Compared to pure metal, metal oxide is more complex as it has bonding varying from nearly ionic to highly covalent and even metallic bond in nature; it comes in different forms with each posing unique compositions, morphologies, structures and physicochemical properties [6]. Different from their bulk form, nanoparticles (NPs) are able to provide a new and promising solution, using their unique features as they have high surface area-to-volume ratio with unique physicochemical properties [7,8,9,10,11,12] widely applied in electronic and solar energy devices, medicinal, environmental remediation, consumer products and catalysis [13,14,15,16,17]. Among the metal oxide NPs, synthesis of CuO NPs are promising as they are cheaper than other Nobel metals [18,19] and can be easily mixed with polymers due to their stability [20]. They are a *p*-type semiconductor in monoclinic structure with the narrow bandgap of 1.2–2.0 eV [21,22]. The CuO NPs exhibit excellent electrical, optical, magnetic phase, antioxidant and antimicrobial properties [2,3,20], which is useful in anti-cancer, biomedical imaging, drug and cellular deliveries, wound healing, and disease treatment, and they act as a heterogeneous catalysis [3,18,23,24]. In recent development, CuO NPs have great potential in various field of applications, which include advanced photocatalytic processes [25], environmental and energy conversion [26], green production of hydrogen, photoelectrochemical water splitting [27], organic pollutants’ photodegradation, generation of solar fuel and photovoltaic devices [28].

The conventional methods in synthesizing CuO NPs include vapor deposition, thermal deposition, radiolysis reduction, thermal decomposition, chemical reduction, hydrothermal, chemical precipitation, solid-state thermal conversion of precursors, electrochemical method, microwave irradiation, sol-gel method, microemulsion, sonochemical process and other combining methods [21,23]; these have been reported by researchers and have resulted in different morphologies, compositions and sizes [14,15,17,29]. Compared with physical and chemical synthesis which have low materials conversion and reaction rates, require high energy, are tedious, non-ecofriendly, technically complex, extremely costly and trendier [6,20,30,31] in process, green synthesis offers a lower-cost, more eco-friendly, simpler, lower energy consuming and minimal chemical using [32,33,34,35,36] way to synthesis NPs. The green-synthesized NPs have longer lifespan compared to conventionally synthesized NPs and can be scaled up easily [20,32,37,38], offering interesting applications in biomedical and related fields [14,15,39]. Green mediated approaches towards the synthesis of CuO nanomaterials by using *Aloe barbadensis* (leaf) [2], *Carica papaya* L. (peel) [22], *Stachys lavandulifolia* [40], *Muntingia calabura* (leaf) [41], *Cedrus deodara* [42] and *Bougainvillea* (flower) [43] were reported recently. The literature survey unveils that there is no report existing on the green synthesis of cupric oxide nanostructures using the plant *Garcinia mangostana* L. Morphology, composition, size, shape and the presence of capping agents in NPs produced are the main challenges in green synthesis as those factors control the NPs’ application in industrial and biomedical purposes [32,44]. Consequently, numerous processing routes should be studied which include the precursors concentrations, reaction conditions (plant extract concentrations, calcination temperatures, calcination times, solvents, etc.) to synthesis CuO NPs in controlling shapes, dimensional morphological features, crystalline and dimensional size with different properties. However, very little is known about the effect of calcination temperatures on morphology, composition, size and shape of CuO nanostructures derived from plant extracts [13,23,45,46].

*G. mangostana* L., commonly called mangosteen, is an endemic evergreen tree species which is native to tropical countries such as Thailand, Indonesia and Malaysia. It belongs to the Clusiacae family [47,48], and mangosteen has been described as the “fruit queen” due to its incomparable flavor and aroma [49,50,51]. The mangosteen contains large amounts of phenolics and antioxidants [52,53,54,55]. Mangosteen leaves especially contain large amounts of 1,5,8-trihydroxy-3-methoxy-2-(3-methylbut-2-enyl) xanthone and 1, 6-dihydroxy-3-methoxy-2-(3-methyl-2-buthenyl)-xanthone [56]. Therefore, mangosteen is reported as having various different medical benefits, such as antioxidant, antifungal, anti-inflammatory, antibacterial, anti-tumor, anti-diabetic, anti-plasmodial, immunity increasing, hepatoprotective functioning, anti-cancer, anti-allergic and anti-leukemic [53,54,55,57,58] properties. The presence of polyphenolic compounds plays an important role in capping, stabilizing and reducing agents in green synthesizing NPs. Herein, we describe a facile and environmentally benign approach for the synthesis of CuO nanoparticles using aqueous extract of *G. mangostana* (mangosteen) leaf as a reducing agent as well as a capping agent. The effect of the calcination temperature (200 °C, 300 °C, 400 °C, 500 °C and 600 °C) on the structure, morphology and optical properties of the green-synthesized CuO NPs was investigated.

## 2. Materials and Methods

### 2.1. Materials

The mangosteen leaves were collected from a neighborhood in Kampar, Malaysia. Copper (II) nitrate trihydrate [Cu(NO_3_)_2_.3H_2_O] was purchased from HmbG (Hamburg, Germany) and used without further purification. All glassware was washed with deionized water and dried in an oven before use.

### 2.2. Preparation of Mangosteen Leaf Extract 

Freshly plucked mangosteen leaves were washed several times with tap water to expel debris and particulate and allowed to dry in an oven at 60 °C for 72 h. Once dried, the leaves were ground into fine powder using a grinder. Then, approximately 5 g of powder was added to 100 mL of deionized water and allowed to heat at 80 °C for 20 min. Upon cooling, leaf extract was filtered through vacuum filtration and reddish-brown filtrate was collected in a 100 mL beaker and immediately used for the synthesis of CuO NPs. 

### 2.3. Synthesis of CuO NPs 

**Scheme 1:**
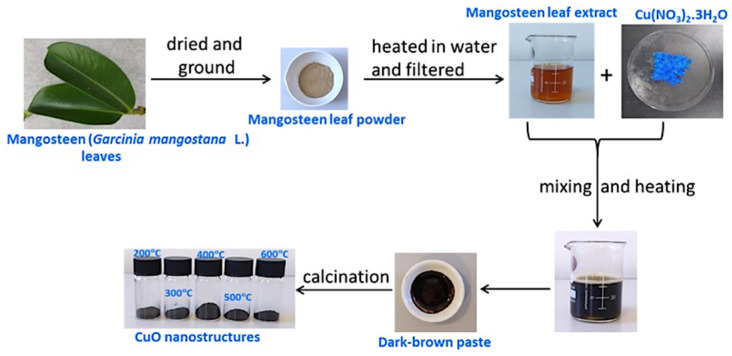
Experimental procedure for green synthesis of CuO nanostructures using aqueous extract of *Garcinia mangostana* L. (mangosteen) leaf.

For the synthesis of CuO NPs, initially, 30 mL of the freshly prepared mangosteen leaf extract was heated and stirred at 70–80 °C. Later, 2 g of Cu(NO_3_)_2_·3H_2_O was gradually added into the hot leaf extract, and a greenish-brown colored solution was formed immediately and was continually heated at 70–80 °C with constant string until the formation of a dark-brown paste. Subsequently, the dark-brown paste was cooled to room temperature, transferred to a ceramic crucible and calcinated at different temperatures (200, 300, 400, 500 and 600 °C) for 2 h using a temperature-controlled muffle furnace to obtain a fine black powder of CuO. Scheme 1 illustrates mangosteen (*Garcinia mangostana* L.) leaf extract-mediated green synthesis of CuO nanostructures at different calcination temperatures.

### 2.4. Characterization of Synthesized CuO NPs

Structural and morphological characterization of as-synthesized CuO NPs were analyzed by various analytical techniques. The absorption spectra were recorded by an UV−visible Spectrophotometer (Thermo Scientific GENESYS 10S). The recombination of electron-hole pairs of the synthesized samples was investigated using photoluminance spectroscopy (Perkin Elmer LS 55 Fluorescence Spectrometer, Waltham, MA, USA) with excitation wavelength at 350 nm in the range 350–600 nm. FTIR studies were carried out at room temperature in the range of 400−4000 cm^−1^ with resolution of 4 cm^−1^ by using KBr pellets in a Perkin Elmer RX1 spectrophotometer. The X-ray powder diffraction patterns were taken in reflection mode with Cu Kα (λ = 1.5406 Å) radiation in the 2*θ* range from 10° to 80° by using a Shimadzu XRD 6000 (Kyoto, Japan) X-ray diffractometer by continuous scanning which was operated at 40 kV/30 mA and 0.02 min^−1^. The X-ray photoelectron spectra was obtained using Perkin Elmer PHI5600 (ULVAC-PHI, Inc, Waltham, MA, USA). A FESEM [JEOL JSM-6710F, Kyoto, Japan combined with energy dispersive X-ray analyzer (X-max, 150 Oxford Instruments)] and HRTEM (JEOL JEM-3010) were used for morphological, microstructural and elemental compositional analysis of synthesized CuO NPs. 

## 3. Results and Discussion

### 3.1. Optical Analysis

**Figure 1:**
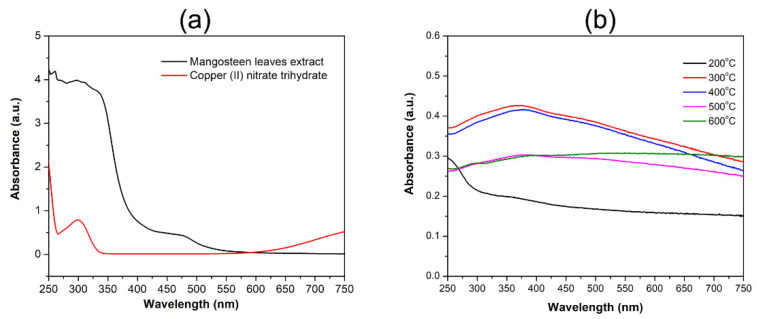
UV-Vis spectrum of the (**a**) mangosteen leaf extract and copper salt and (**b**) *G. mangostana* L. leaf extract-mediated synthesized CuO NPs at different calcination temperatures.

**Figure 2:**
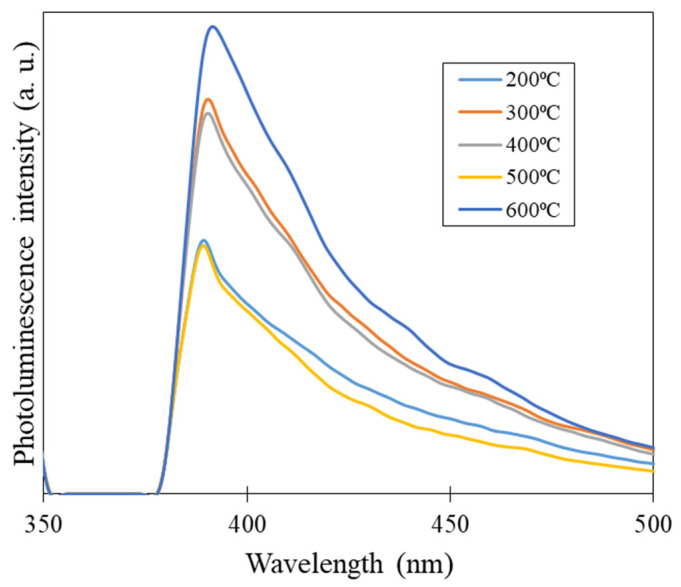
PL spectrum of the *G. mangostana* L. leaf extract-mediated synthesized CuO NPs at different calcination temperatures.

**Figure 3:**
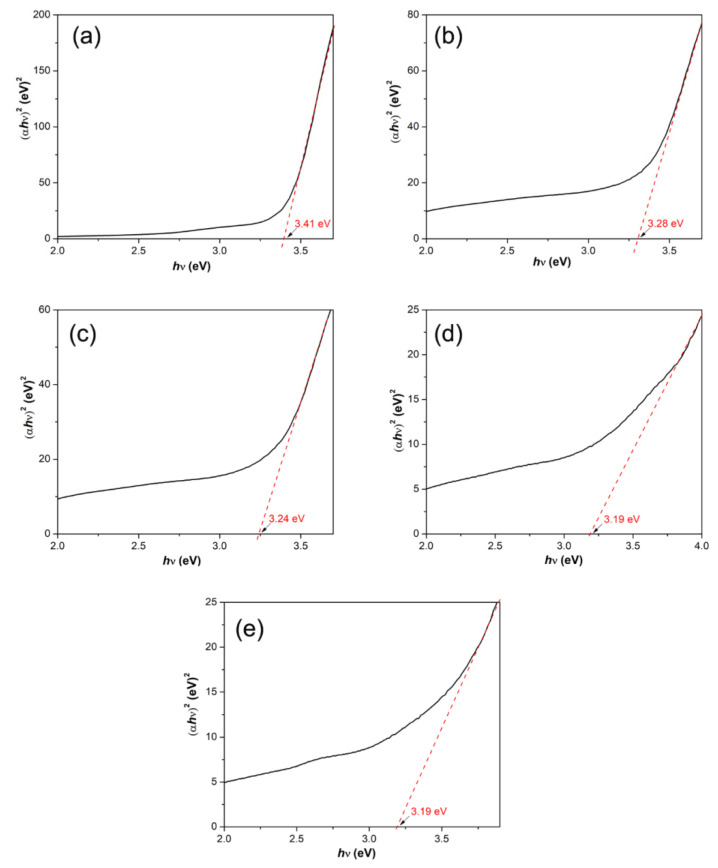
Energy bandgap of the *G. mangostana* L. leaf extract-mediated synthesized CuO NPs calcinated at (**a**) 200 °C, (**b**) 300 °C, (**c**) 400 °C, (**d**) 500 °C and (**e**) 600 °C using Tauc plot approach.

Figure 1 depicts the UV-Vis spectrum of the synthesized CuO NPs at different calcination temperatures with mangosteen leaf extract and copper salt. Meanwhile, Figure 2 and Figure 3 show the PL spectrum and energy bandgap, respectively, of synthesized CuO NPs at varied calcination temperatures. The formation of a broad absorption peak of mangosteen leaf aqueous extract was observed at 439 nm due to the π → π* transition of phytochemicals, whereas 295 nm for copper nitrate. During the synthesis of CuO NPs, the leaf extract color change from light-brown to dark brown was observed after adding copper salt, indicating the reduction of Cu^2+^ to CuO with the presence of phytochemicals in the mangosteen leaf extract. Similar observations were reported from green to dark brown and bluish to dark green in green synthesizing CuO NPs using *Cedrus deodara* leaf [42] and *Bougainvillea* flower [43] extracts. The color changes before and after green synthesis of CuO NPs were due to the surface plasmon resonance (SPR) [59,60,61]. The presence of phytochemicals in plant extract generates the electrons and causes the reduction of copper salt and conversion into CuO NPs [3]. As a result, the SPR resulted from the electron resonant oscillating at the conduction band initiated by incident electromagnetic radiation [2,22,40,41] at specific wavelength [62]. Thus, the absorption peak at 344–522 nm from the current study indicates the formation of CuO NPs.

The recombination of photogenerated electron-hole pairs of the samples was investigated using PL. The PL spectrum of the samples shows a peak centered around 390 nm which corresponds to band-edge emission [63]. This value coincides with previously reported literature on copper oxide nanoparticles [64]. High intensity of PL indicates great recombination of charge carriers, but low PL intensity suggests the maximum charge carriers separation which is useful in photodegradation [65]. A calcination temperature-dependent shift of the absorption peak was observed following the increase in calcination temperature in synthesizing CuO NPs. A red shift of the absorption peak was observed with increasing calcination temperature, suggesting the aggregation and larger size of NPs formed at higher calcination temperature with lesser strain [66,67]. The red shift of the absorption peak might be attributed to the capability donation of free electrons to the copper vacant orbital facilitated by the electron transition [68] at higher calcination temperature and resulting in a lower energy bandgap [44,66]. The broad peak extending from 259–462 nm suggests that the distribution of particle size is large [7]. The broadest absorption peaks were observed for synthesized CuO NPs calcinated at 600 °C, representing polydispersion [69] of NPs and indicating the NPs production was in various sizes [8]. Thus, the optical properties of synthesized CuO NPs are greatly affected by the temperature, size and morphology [14].

Table 1 shows the relationship between energy bandgap ($E_{g})$ and calcination temperature. The CuO is known to be a direct-allowed semiconductor [70]. The energy band gap of synthesized CuO NPs was expressed in eV and calculated using Tauc’s approach with the following formula:

(1) $$\alpha hv=A{\left(hv-E_{g}\right)}^{n}\;$$

where, *h* is Planks constant (6.626 × 10^−34^ Js), *n* is exponential factor for electronic transition (*n* = ½, for indirect band, *n* = 2, for direct band) and *α* is absorption coefficient. Lower energy bandgap at higher calcination temperature might be due to the interaction in xanthones’ functional group of mangosteen leaf extract with precursors in co-precipitation of CuO NPs that resulted in increasing crystallinity. The increase in crystallinity is reported to increase the energy of the electron and reduce the energy bandgap [23,71]. This phenomena is called as quantum size effect [21]. The reduction in energy bandgap with increasing calcination temperature is also due to the presence of surface dangling bonds surrounding the crystallites. Under heating treatment, crystallization takes place and results in dangling bonds. The crystallites break down under higher calcination temperature and cause the increase in the number of surface dangling bonds. As a result, concentration of localized states in the band structure and width increases gradually, thereby reducing the energy bandgap [66]. Both reasons are proven by the red shifting of the absorption peak [63,64] and, subsequently, the energy bandgap was reduced with the increasing calcination temperature. The lower and decreasing trend of the energy bandgap was reported in *Fumaria indica* extract mediated-CuO NPs at elevated calcination temperature (100, 300, 600 and 900 °C) for 2 h [23].

### 3.2. FTIR Analysis and Functional Groups Determination

**Figure 4:**
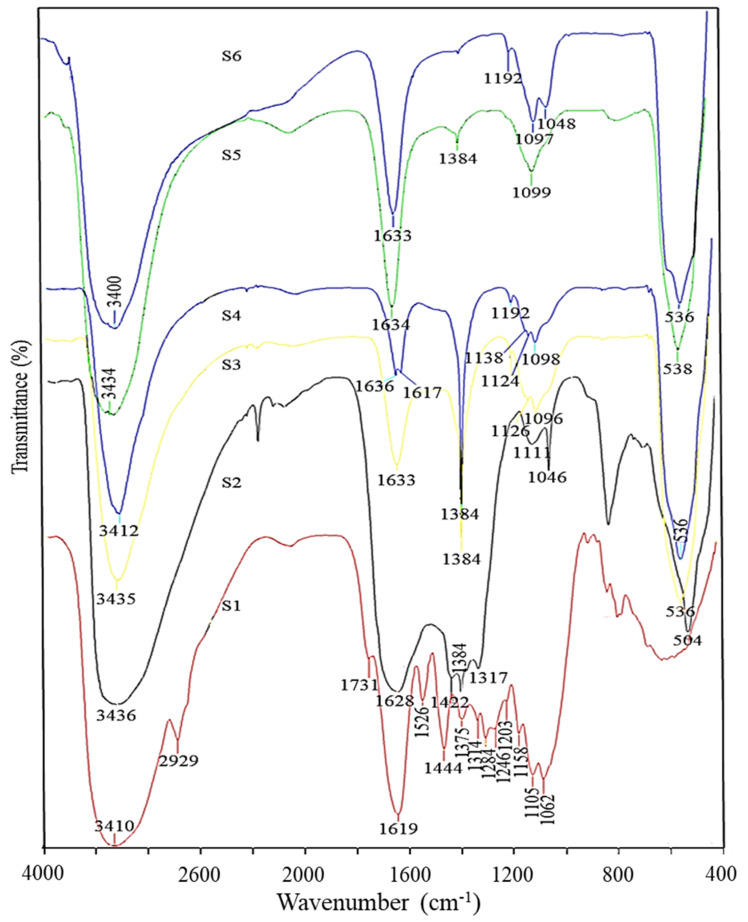
FTIR spectrum for S1 = mangosteen leaf extract, S2 = CuO NPs calcinated at 200 °C, S3 = CuO NPs calcinated at 300 °C, S4 = CuO NPs calcinated at 400 °C, S5 = CuO NPs calcinated at 500 °C and S6 = CuO NPs calcinated at 600 °C.

FTIR spectra were recorded to determine the potential functional groups of phytochemicals in mangosteen leaf extract that are responsible for the formation of CuO nanostructures and shown in Table 2. Figure 4 shows the FTIR spectra of mangosteen leaf extract and CuO nanostructures calcinated at different temperatures. Compared with mangosteen leaf extract, most of the peaks were disappeared in synthesized CuO NPs, which included 2929, 1731, 1526, 1444, 1314, 1284 and 1246 cm^−1^ (Table 2 and Figure 3). The disappearance of peaks in synthesized CuO NPs might be due to the fact that the phytochemicals are mainly responsible for reducing the copper ions [32,72]. This might also be applied for the less intense 1384 cm^-1^ peak for the synthesized CuO NPs calcinated under 500 °C. In addition, some of the peaks were slightly shifted in synthesized CuO NPs. Compared with mangosteen leaf extract, the peak at 3410 cm^−1^ was shifted to 3400–3436 cm^−1^, 1619 cm^−1^ was shifted to 1617–1636 cm^−1^, 1375 cm^−1^ was shifted to 1384 cm^−1^, 1203–1105 cm^−1^ were shifted to 1192–1096 cm^−1^ and 1062 cm^−1^ was shifted to 1046–1048 cm^−1^, respectively, in synthesized CuO NPs. The shifting of peaks indicate the involvement of mangosteen leaf extract phytochemicals in reducing, capping and stabilizing agents [32,54,66,73,74] in synthesized CuO NPs through electrostatic and steric stabilization [75]. Meanwhile, the peaks remain unchanged, showing the suggested phytochemicals functional groups were responsible for stabilizing NPs [32]. The presence of the peculiar peak at 2300–2350 cm^−1^ in sample S2 might be due to the contamination from phytochemicals on the synthesized CuO NPs surface calcinated at low calcination temperature. Nevertheless, a sharp and intense peak located at 504–536 cm^−1^ only appeared in synthesized CuO NPs at higher calcination temperatures, indicating the presence of copper-oxygen bond vibration as reported between 420–613 cm^−1^ in other studies [2,18,22,24,40,41,42,43]. The absorption peak of metal oxides and hydroxides NPs are commonly located at the fingerprint region (wavenumber below 1000 cm^−1^) due to interatomic vibrations [76]. Slight changes in Cu-O bond vibration at different calcination temperature are caused by the interaction with plant extract functional groups [8]. 

### 3.3. Crystalline and Structural Analysis

**Figure 5:**
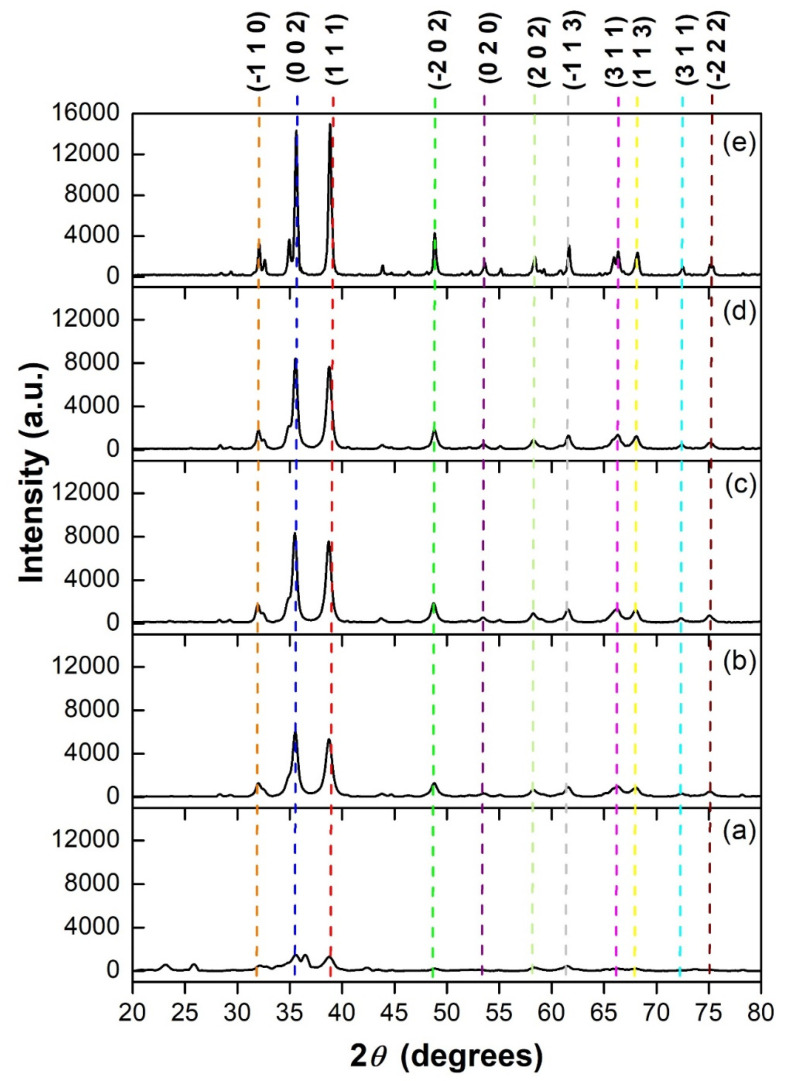
XRD pattern of *G. mangostana* L. leaf extract-mediated synthesized CuO NPs at (**a**) 200 °C, (**b**) 300 °C, (**c**) 400 °C, (**d**) 500 °C and (**e**) 600 °C calcination temperatures.

Table 3 shows the unit cells, crystalline size, dislocation density and micro strain, while Figure 5 shows the XRD patterns, respectively, for synthesized CuO NPs calcinated at different temperatures. The synthesized CuO NPs were in in good agreement with ICDD 00-045-0937. All synthesized CuO NPs were in monoclinic system which are reported in other studies [18,22,23,40,42]. The C*2*/*c* space group in all synthesized CuO NPs were the same as reported in the Joya et al. study in chemically synthesized CuO NPs calcinated at 650 and 800 °C [77]. The tenorite phase was only observed in all synthesized CuO NPs in the current study. Tenorite is a CuO mineral that consists of copper and oxygen. Pure tenorite can be obtained by calcining the copper minerals at high temperature [78]. However, it was only observed in chemically synthesized CuO NPs calcinated at 550 and 1000 °C in the Habibi and Karimi [70] and Ratnawulan et al. [78] studies, respectively. The well-defined, high-intensity and narrower diffraction peaks indicated the synthesized CuO NPs were well-crystalline [18,22,24] as the organic materials are removed during the calcination process in improving the crystallinity [79]. More distinct XRD peaks without any impurity peaks show the purity of synthesized CuO NPs [41] with increasing calcination temperatures [80] due to the decomposition of impurities [81]. This phenomena also indicates the increase in crystalline size at higher calcination temperature [80]. The intensities of (002) and (111) peaks were stronger than other peaks, showing that they are the preferential crystal planes of NPs [67]. The crystalline size was calculated using Debye-Scherrer’s formula:

(2) $$D=\frac{0.94\lambda }{\beta cos\theta }\;$$

where, *D* is the crystalline size of NPs, *λ* is X-ray wavelength, *β* is full width half maximum (FWHM) of the peak and *θ* is Bragg angle. The crystalline size of synthesized CuO NPs increased from 12.78 to 28.17 nm with increasing calcination temperatures due to the tendency for minimization of the interfacial surface energy [80]. Additionally, the diffusion atoms were increased at elevated calcination temperature. The increase in atom diffusion resulted in the increasing formation of nuclei with the grain boundary separated by pores. When the calcination temperature constantly increased, the grain boundary disappeared, and the crystalline size increased [67,78]. Also, the agglomeration, re-crystallization, aggregation and growth of particles at higher temperature are the reasons for the increase in crystalline size at elevated calcination temperature [17,66]. This suggests that the stability of crystals was improved at higher calcination temperatures [23] and shows a strong relationship between average crystalline size of CuO NPs with calcination temperatures [80]. The above-mentioned result was similar reported synthesized CuO NPs in other studies with increasing calcination temperature, either using chemical [77] or green synthesis [13,23,46]. The dislocation density of the synthesized CuO NPs was calculated as follows:

(3) $$\delta =\frac{1}{D^{2}}\;$$

where, *δ* is dislocation density of NPs, and *D* is NPs crystalline size. The dislocation density was found higher at lower calcinated CuO NPs as the number of interfaces in a given volume was greater with smaller crystalline size. The same trend was reported in the Kayani et al. study in chemically synthesized CuO NPs calcinated at 400 and 1000 °C [82]. The micro strain of the synthesized CuO NPs was calculated as follow:

(4) $$\varepsilon =\frac{\beta cos\theta }{4}\;$$

where, *ε* is the micro strain of NPs, *β* is FWHM of the peak and *θ* is Bragg angle. The micro strain is able to give information about the defects present in the lattice. Lower micro strain was reported at higher calcinated CuO NPs due to more defects being removed at elevated calcination temperature [66].

**Figure 6:**
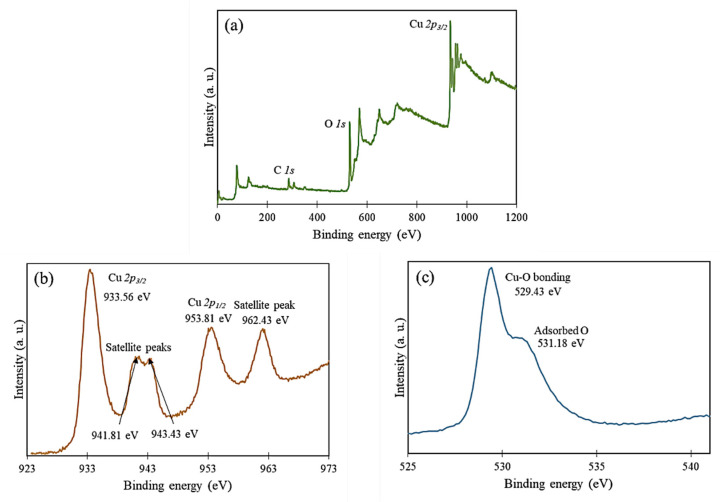
XPS measurement of (**a**) mangosteen leaf extract-mediated CuO NPs, (**b**) high-resolution Cu *2p* peaks and (**c**) high-resolution O *1s* peaks.

The purity and chemical composition of the mangosteen leaf extract mediated-CuO NPs calcinated at 500 °C was investigated by XPS and shown in Figure 6. As the high-resolution XPS spectrum shows in Figure 6b, the Cu 2*p* core level shows two peaks at 933.56 and 953.81 eV and indicates the detection of Cu *2p_3/2_* and Cu *2p_1/2_*, respectively, which correlate to Cu^2+^ valency state. The 20.25 eV difference of both peaks matches with other CuO NPs. The appearance of both satellite peaks confirms the presence of the d^9^ Cu^2+^ fingerprint caused by the relaxation circumstance of the strong alignment interaction in the final state. The remaining peaks located at higher binding energy (941.81, 943.43 and 962.43 eV) than main spin-orbital components reveal the shake-up satellites of Cu^2+^ peaks. The high-resolution XPS spectrum of O *1s* was shown in Figure 6c. The O^2-^ in Cu-O bonding was located at lower binding energy (529.43 eV) compared to adsorbed O (531.18 eV) on the CuO surface. From the XPS measurement, therefore, the presence of Cu_2_O and Cu(OH)_2_ were excluded in the green-synthesized CuO NPs calcinated at 500 °C, as there would be no satellite peaks for Cu *2p_3/2_* and Cu *2p_1/2_* of Cu^2+^ [22,41]. Overall, the XPS measurement confirms the mangosteen leaf extract-mediated CuO NPs calcinated at 500 °C are in single phase without the presence of impurities.

### 3.4. Morphologies and Elemental Analysis

**Figure 7:**
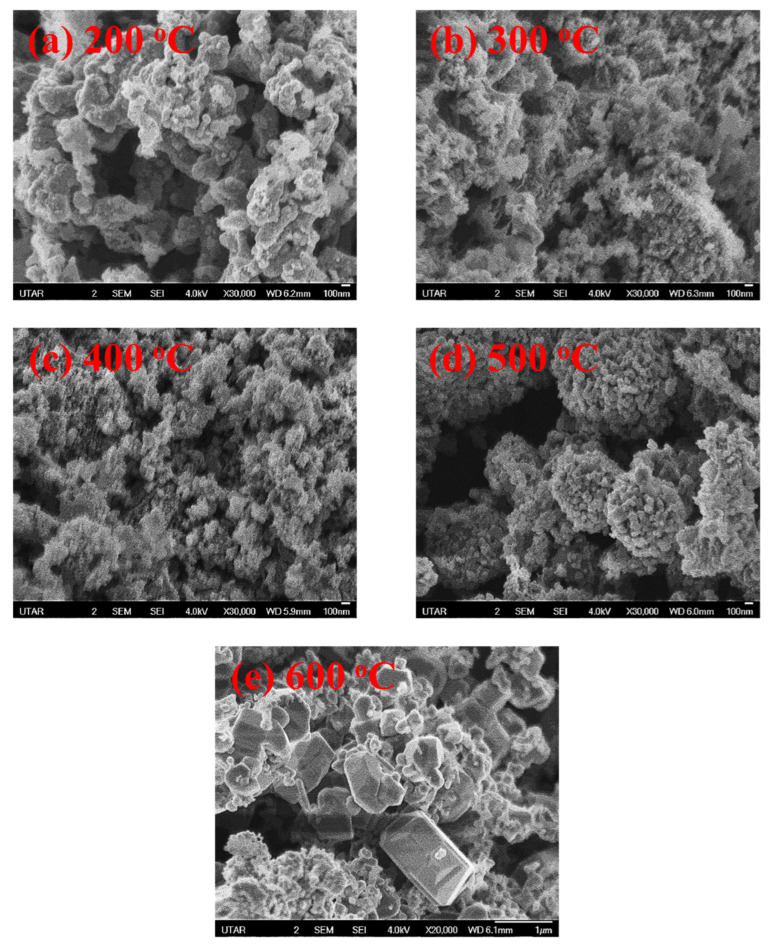
Morphologies of *G. mangostana* L. leaf extract-mediated synthesized CuO NPs calcinated at (**a**) 200 °C, (**b**) 300 °C, (**c**) 400 °C, (**d**) 500 °C and (**e**) 600 °C under SEM.

**Figure 8:**
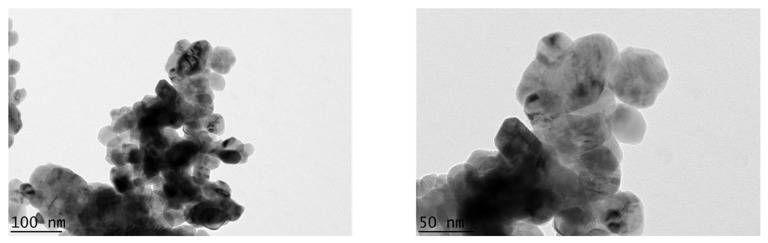
Morphologies of *G. mangostana* L. leaf extract-mediated synthesized CuO NPs calcinated at 500 °C under TEM.

Figure 7 shows the morphologies of *G. mangostana* L. leaf extract-mediated synthesized CuO NPs at different calcination temperatures. FESEM images confirmed the nanostructure of synthesized CuO NPs with agglomeration. The higher the calcination temperature, the larger the particle size [77] as the mangosteen leaf extract-mediated CuO NPs average particle size was increased from 50.0 nm to 458.3 nm at elevated calcination temperature. The synthesized CuO NPs were agglomerated due to high surface energy, surface area, surface tension, surface reactivity [2,9,22,74,83,84], the viscous nature of plant extract [24], attraction among the NPs [30,74,85] and oxidation of metal oxide NPs [42]. Isotropic aggregation usually leads to the formation of a spherical shape, and this aggregation occurs at the isoelectric point region [81]. As a result, the synthesized NPs stick strongly together with considerable affinities to form asymmetrical clusters [84], more or less in spherical shape with rough surface [34,81]. Thus, quasi-spherical (400 °C) and spherical shapes (500 °C) were observed in synthesized CuO NPs. The above-mentioned observations are similar to chemically synthesized CuO NPs in other studies calcinated between 300–1000 °C [17,77,80], respectively. Nonetheless, mixed morphology was observed in sonochemical synthesized CuO NPs in the Saravanan and Sivasankar study calcinated at 400 °C [17]. In green synthesis, spherical CuO NPs were reported using *Aerva javanica* leaf [18], *Punica granatum* peel (24) and *Stachys lavandulifolia* flower [40] extracts, respectively, which were similar to the current study that synthesized CuO NPs calcinated at 500 °C. The formation of nanoflake CuO NPs at 200 and 300 °C in the current study shows the progressively forming CuO NPs [45] when calcination temperature is increasing. The formation of bulky particles in synthesized CuO NPs calcinated at 600 °C was due to the disappearance of the grain boundary area as the growing grains were disturbing and decreasing the crystal surface energy during the aggregation under calcination, which caused the pore volume to decrease, leading to compact shrinkage when calcination temperature increases [80,81]. A similar observation was reported in chemically synthesized CuO NPs calcinated at 700 °C [80]. However, fairly uniform spherical shapes were observed in the Fardood and Ramazani study using black tea extract to synthesis CuO NPs calcinated at 600 °C [45]. The agglomeration of synthesized CuO NPs at 600 °C might be due to the removal of carbonized materials [86] which is supported by the Fardood and Ramazani study using coffee powder extract to synthesize CuO NPs [13]. Thus, the SEM images revealed that the coarsening and coalescence occurred due to the changes in calcination temperature and resulted in NP morphology alteration [23,83]. The particle morphology, size and shape of green-mediated CuO NPs calcinated at 500 °C were also determined using TEM at different magnifications as shown in Figure 8. TEM micrographs shows that the green-mediated CuO NPs were in spherical and quasi-spherical structure with the particle size in the range of 15.0–50.0 nm.

**Figure 9:**
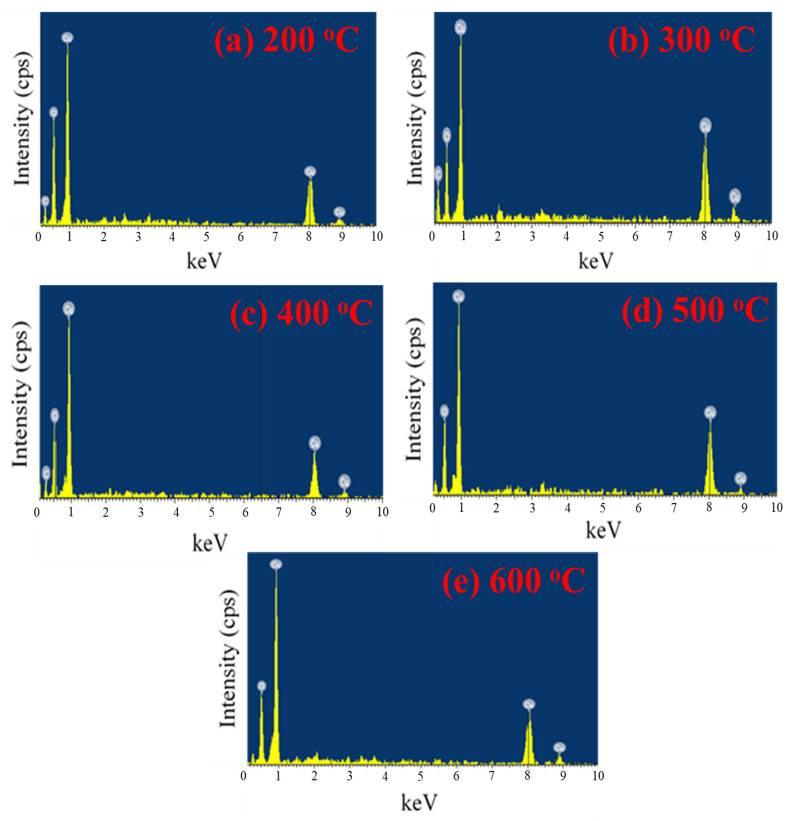
EDX spectrums of *G. mangostana* L. leaf extract-mediated synthesized CuO NPs calcinated at (**a**) 200 °C, (**b**) 300 °C, (**c**) 400 °C, (**d**) 500 °C and (**e**) 600 °C.

Figure 9 shows the *G. mangostana* L. leaf extract-mediated synthesized CuO NPs EDX spectrums at different calcination temperatures. The strong signal with high intensity of copper and oxygen elements shown in EDX spectrums indicates the formation of CuO NPs. At low calcination temperatures (200, 300 and 400 °C), a weak signal of carbon appeared as an impurity in the synthesized CuO NPs due to the slow reduction of attached phytochemicals [87] as reducing, stabilizing and capping agents in green synthesizing NPs [10,59,73,82]. Also, the presence of carbon might be caused by the contamination from environment or substrate or adsorption of phytochemicals on the surface of NPs [19,68]. A similar result was found in the Ruda et al. study in synthesizing CuO NPs using precipitation method calcination under 100, 250 and 400 °C [19]. Additionally, Siddiqi and Husen mentioned that heating at 400 °C causes burning of any organic matters and should leave only soot or carbon as impurities [20]. However, this is not a major problem as only one or two atoms remained to show less interaction [87]. Subsequently, the weak signal of carbon disappeared and only copper and oxygen remained at high calcination temperatures (500 and 600 °C), revealing that the phytochemicals on the CuO NPs eliminate completely as CO_2_, H_2_O or nitrogen oxide (NO_x_). A similar result was obtained in the Sharma et al. and Selvanathan et al. studies using *Aloe barbadensis* leaf [2] and *Muntingia calabura* leaf [41] extracts, respectively. The absence of impurities shows that the synthesized CuO NPs calcinated at 500 and 600 °C were nearly stoichiometric [88]. All the synthesized CuO NPs had the copper atom located at 0.90, 8.05 and 8.90 keV, respectively, while the oxygen atom was located at 0.50 keV. Nonetheless, carbon was only found as an impurity in synthesized CuO NPs calcinated at low calcination temperatures located at 0.25 keV. The above-mentioned locations of copper, oxygen and carbon atoms were confirmed by the Phang et al. and Veisi et al. studies using *Carcinia papaya* L. peel [22] and *Stachys lavandulifolia* flower [40], respectively. 

### 3.5. Comparison with Other Studies

Table 4 shows the comparison of the current study’s green-mediated CuO NPs with other studies at various calcination temperature using different plant aqueous extract. Compared with others, the current study has the largest average particle size (50.0–458.3 nm). This might be due to the high viscosity of mangosteen leaf aqueous extract. The energy bandgap in the current study is within 3.41–3.23 eV, which is in-between the reported studies. Additionally, the current study CuO NPs have the smallest average crystalline size (12.78–28.17 nm) compared to other studies. This shows that the phytochemical content in mangsoteen leaf aqueous extract has better capability in capping and stabilizing CuO NPs crystalline in nano-range. 

## 4. Conclusions

The CuO nanostructures were successfully synthesized using *G. mangostana* L. leaf extract and the effect of calcination temperature on the morphology, crystalline size, purity and optical properties of these nanostructures was investigated. Red shifting of the absorption peak and decreasing in the energy bandgap of synthesized CuO NPs indicate larger particle size was formed with increasing calcination temperature. The PL spectrum shows that the mangosteen leaf aqueous extract-mediated CuO NPs have the greatest charge carriers separation calcinated at 500 °C. The phytochemicals of *G. mangostana* L. leaf extract involved in reducing, capping and stabilizing CuO NPs were shown in FTIR spectrum. The sharper and more intense peak located at 504–536 cm^−1^ at elevated calcination temperature indicates the presence of the copper-oxygen vibration bond. The XRD patterns showed the tenorite phase, monoclinic system and C2/*c* space group in all synthesized CuO NPs. The increase in calcination temperature increased the sharpness and intensity of the XRD diffraction peaks, revealing that the increase in crystalline size from 12.78 to 28.17 nm resulted in the decrement in dislocation density and micro strain. Through XPS measurement, the mangosteen leaf extract-mediated CuO NPs calcinated at 500 °C is confirmed in single phase without the presence of impurities. Although calcinated at different temperatures, the synthesized CuO NPs were more or less in spherical in structure as observed in FESEM micrographs which is similar with the observation found in TEM micrographs for green-synthesized CuO NPs calcinated at 500 °C. The presence of copper and oxygen elements was confirmed by EDX while the carbon element only appeared as impurity at lower calcinated CuO NPs. Conclusively, calcination temperatures strongly influenced the crystalline size, morphology, purity, and optical properties of *G. mangostana* L. leaf extract-mediated synthesized CuO nanostructures which may influence their performance in various applications such as a photocatalyst in wastewater treatment, antimicrobial agent in healthcare and so on.

## Figures and Tables

**Table 1:** Relationship between energy bandgap and calcination temperature.

Calcination Temperature (°C)	Energy Bandgap (eV)
200	3.41
300	3.28
400	3.24
500	3.19
600	3.19

**Table 2:** FTIR peak assignment of CuO nanostructures calcinated at different temperatures and mangosteen leaf extract.

Mangosteen Leaf Extract	200 °C	300 °C	400 °C	500 °C	600 °C	Functional Groups
Peaks (cm^−1^)	Peaks (cm^−1^)	Peaks (cm^−1^)	Peaks (cm^−1^)	Peaks (cm^−1^)	Peaks (cm^−1^)	
3410	3436	3435	3412	3434	3400	O-H stretching; N-H stretching
2929	-	-	-	-	-	C-H stretching *sp*^3^, O-H stretching
1731	-	-	-	-	-	C=O stretching
1619	1628	1633	1636, 1617	1634	1633	C=O stretching; C=C vibration; N-H bend
1526	-	-	-	-	-	C-C aromatics bond
1444	1422	-	-	-	-	CH_2_- bending_;_ C-C aromatics stretching
1375	1384	1384	1384	1384	-	C-C aromatic stretching; N-O symmetric stretching
1314	1317	-	-	-	-	-CH_3_ bending
1284	-	-	-	-	-	C-O-C stretching; C-O stretching; C-OH vibration
1246, 1203, 1158, 1105	1111	1190, 1126, 1096	1192, 1138, 1124, 1098	1099	1192, 1097	C-O-C stretching; C-O stretching
1062	1046	-	-	-	1048	C-O ester
-	504	536	536	538	536	Cu-O bond vibration

**Table 3:** Unit cells, crystalline size, dislocation density and micro strain of *G. mangostana* L. leaf extract-mediated synthesized CuO calcinated at different temperatures.

Calcination Temperature (°C)	Unit Cells				Average Crystalline Size (nm)	Dislocation Density (10^14^ cm^−1^)	Micro Strain (10^−4^)
	*a* (Å)	*b* (Å)	*c* (Å)	*β* (^o^)			
200	4.6797	3.4314	5.1362	99.262	12.78	61.26	3.40
300	4.6797	3.4314	5.1362	99.262	14.04	50.70	2.64
400	4.6820	3.4240	5.1270	99.420	18.32	29.80	1.97
500	4.6853	3.4257	5.1303	99.549	19.88	25.31	1.82
600	4.6900	3.4200	5.1310	99.540	28.17	12.60	1.26

**Table 4:** Comparison of plant-mediated CuO NPs at different calcination temperatures with other studies.

Plant Extract	Calcination Temperature (°C)	Energy Bandgap (eV)	Average Crystalline Size (nm)	Average Particle Size (nm)	Morphology	References
Coffee powder	500, 600, 700, 800	-	24.72–35.65	24.72–35.65	Fairly uniform shape with narrow size distribution	[13]
*Fumaria indica*	100, 300, 600, 900	1.82–1.46	14.90–79.29	24.59–58.92	Cracking of cluster and forming flake at elevated calcination temperature	[23]
Commercial black tea powder	500, 600, 700, 800	N/A^1^	22.30–38.70	22.3–38.7	Fairly uniform shape with narrow size distribution	[42]
*Prunus amygdalus* pericarp	400, 500, 600	4.59	11.76	-	More spherical at elevated calcination temperature	[43]
*G. mangostana* L. leaf	200, 300, 400, 500, 600	3.41–3.23	12.78–28.17	50.0–458.3	More spherical and larger size at elevated calcination temperature	Current study

## Data Availability

The data presented in this study are available upon request from the corresponding author.
